# Diet-induced obesity alters myeloid cell populations in naïve and injured lung

**DOI:** 10.1186/s12931-016-0341-8

**Published:** 2016-03-08

**Authors:** Anne M. Manicone, Keqin Gong, Laura K. Johnston, Matthew Giannandrea

**Affiliations:** Center for Lung Biology, Division of Pulmonary and Critical Care Medicine, University of Washington, 850 Republican Avenue, Seattle, WA 98115 USA; Northwestern University, Chicago, IL USA

**Keywords:** Diet-induced obesity, Neutrophils, Macrophages, LPS, Acute lung injury, Inflammation

## Abstract

**Background:**

There are pulmonary consequences to obesity, including increased prevalence of asthma, greater susceptibility to influenza, and possibly reduced susceptibility to lung injury. Although it is well established that obesity is associated with alterations to the immune system, little is known about obesity-associated changes to pulmonary immune cells.

**Objectives:**

We hypothesized that obesity would alter the inflammatory milieu in the unchallenged lung and circulation; thereby contributing to altered susceptibility to lung injury.

**Methods:**

We used a murine model of diet-induced obesity and evaluated bone marrow and blood leukocytes at 3 months, and pulmonary leukocytes at 3 and 6 months for changes in their adhesion and chemokine receptors, markers of activation states, and cell numbers. We also evaluated the inflammatory response to LPS in obese mice.

**Results:**

In the lung, diet-induced obesity was associated with increased leukocyte numbers over-time. Adhesion receptors were increased in a cell- and site-specific fashion, and there was an evolution of macrophage and neutrophil polarization toward M1 and N1, respectively. After LPS-challenge, obesity was associated with increased neutrophil recruitment to the lung with impaired migration into the alveolar space. Associated with these changes, obesity increased LFA-1 and ICAM-1 neutrophil expression and altered CXCL1 gradients.

**Conclusion:**

Our results highlight the effects of diet-induced obesity on the murine blood and lung leukocyte populations, including increases in adhesion receptor expression that may contribute to altered recruitment or retention within the lung. Translation of these findings to people with obesity will be critical for determining the basic inflammatory underpinnings of pulmonary disease susceptibility.

**Electronic supplementary material:**

The online version of this article (doi:10.1186/s12931-016-0341-8) contains supplementary material, which is available to authorized users.

## Background

The prevalence of obesity, defined as a body mass index (BMI) ≥ 30 kg/m^2^, is of epidemic proportions, affecting 16 % of children and 32 % of adults in the United States [1]. Obesity is associated with adverse outcomes and increased morbidity in many disease processes, such as diabetes, hepatitis, and cardiovascular disease [2, 3]. However, recent data suggest that mortality related to acute lung injury is not increased in the people with obesity, and in fact, may be lower with increasing BMI [4–6]. Although obesity is associated with elevated cytokine levels at baseline, obese patients with acute lung injury (ALI) have lower levels of several proinflammatory cytokines and reduced markers of epithelial cell injury compared to non-obese ALI patients, suggesting that obesity may alter immune function in critical illness and lung injury [7]. The mechanisms by which obesity modifies these effects are unknown, and we hypothesized that diet-induced obesity alters the inflammatory cell populations in the lung contributing to susceptibility to some lung diseases and resistance to others.

Obesity induces a state of low-grade chronic inflammation (also referred to as “metaflammation”), and it does so via multiple pathways. Adipose tissue serves to clear circulating triacyglycerol (TAG), thereby inhibiting the release of free fatty acids. In obesity, the adipocyte is saturated with TAG, resulting in increased circulating TAG and fatty acids, the latter of which is a ligand for the pattern recognition receptor, Toll-like receptor-4 (TLR4) [8]. Adipose tissue also secretes adipocytokines (such as leptin, resistin), and cytokines, among other factors, that may regulate local and systemic inflammatory responses [9]. In obese states, there is increased macrophage recruitment to adipose tissue with progressive repolarization from M2 to M1 cells [10–12], increased systemic proinflammatory mediators, and a leukocytosis with elevated lymphocytes, neutrophils and monocytes reported in both obese children and adults [13]. Hence, obesity can be considered a chronic inflammatory state. To date, the effects of obesity on the naïve leukocytes populations in lung are not well defined. We evaluated the effects of diet-induced obesity on leukocyte populations in the lung at 3 and 6 months on a high fat diet. We report significant changes associated with leukocyte populations, adhesion and chemokine receptor expression, and markers of macrophage polarization that evolve over time. We also report on the differential leukocyte recruitment to the lung post-LPS challenge, with a dramatic increase in neutrophil sequestration to the lung in obese mice with impaired cell migration into the alveolar space. These studies highlight the profound alterations of the pulmonary immune cells associated with obesity, and future studies are needed to translate these findings to human pulmonary disease.

## Methods

### Animals

Age- and gender- matched C57BL/6J mice were used for all experiments. At 7–9 weeks, mice were place on a Western diet (HFD; TD.88137; Harlan Laboratories, Indianapolis, IN) or standard chow diet (LFD; 5053; Lab Diet, St. Louis, MO) for 12–24 weeks. Naïve mice were maintained on LFD or HFD prior to harvest. LPS-treated mice received 80 μg of *E. Coli* LPS 011:B4 (Sigma-Aldrich, St. Louis, MO) in 50 ml PBS via oropharyngeal aspiration while sedated with isoflurane. Mice were euthanized with Beuthanasia-D (Intervet, Millsboro, DE) prior to blood and tissue collection. The University of Washington Office of Animal Welfare approved all animal protocols.

### Blood collection

In naïve mice, blood was collected via cardiac puncture and anticoagulated using 4 % EDTA (Fisher Scientific, Hanover Park, IL). An equal volume of blood per mouse (400 μl) was lysed using RBC lysis buffer per manufacture’s protocol (eBioscience, San Diego, CA). This lysis protocol was repeated to remove remaining erythrocytes. After RBC lysis, pelleted cells were resuspended in an equal volume of FACS buffer (PBS, 0.5 % EDTA).

### Bone marrow collection

In naïve mice, bone marrow from the femur was harvested by brief centrifugation under sterile technique. The cell pellet was resuspended in 500 μl PBS, and erythrocytes were lysed using RBC lysis buffer (eBioscience). After lysis, cells were resuspended in FACS buffer.

### Broncho-alveolar lavage

In LPS-treated mice, the trachea was cannulated with an angiocatheter. Broncho-alveolar lavage was performed with three serial instillations of lavage buffer (PBS, 0.5 % EDTA) (total volume 2.5 ml). BAL cells were pelleted and re-suspended in RPMI + 10 % FBS, and approximately 50,000 cells placed on slides using a cytospin. Cells were stained with Differential quik (VWR, Radnor, PA), and a manual differential was performed on 100 cells. In separate experiments, BAL cells were re-suspended in FACS buffer and processed for FACS. BAL fluid total protein was measured using a Bradford assay (Bio Rad, Hercules, CA).

### Lung tissue processing

To isolate pulmonary leukocytes from naïve mice, the pulmonary vasculature was perfused with 10 ml cold PBS after blood collection via cardiac puncture. The perfused, non-lavaged lungs were then dissociated mechanically with scissors and incubated with Liberase TM (1 mg/ml; Roche, Indianapolis, IN) and DNAase I (1 mg/ml; Sigma Aldrich, St. Louis, MO) for 10 min at 37 °C. Lung digests were filtered through a 70-μm nylon cell strainer (Becton Dickinson, Franklin Lakes, NJ) and erythrocytes removed using RBC lysis Buffer. Cells were re-suspended in FACS buffer.

In LPS experiments, mice underwent broncho-alveolar lavage prior to lung harvesting. Mice had their non-perfused, lavaged lungs inflated with 10 % formalin, paraffin-embedded, and processed for H&E staining. Lungs (all 5 lobes) were scored for an average of severity of inflammation (0 = none, 1 = mild, 2 = moderate, 3 = severe) and distribution (0 = none; 1 = focal; 2 = multifocal; 3 = extensive). In separate experiments, LPS-treated mice had both vasculature perfusion and broncho-alveolar lavage prior to collection of the lung for mechanical and enzymatic dissociation as described above. Cells from these experiments were processed for FACS as below.

### Cell processing for FACS

Blood, bone marrow, BAL, and lung cells were counted using an automated cell counter (Nexcelcom Bioscience, Lawrence, MA). Cells were incubated with Fc block (anti-mouse CD16/CD32; eBioscience) in 100 μl of FACS buffer (PBS, 0.5 % EDTA) followed by the antibody panels below. Labeled cells were analyzed using BD FACSCanto RUO (BD Biosciences, San Jose, CA) and the FlowJo data analysis software (Ashland, OR). Experiments represent an average of *n* = 5/group with up to 3 replicates. Antibodies used included: anti-mouse CD62L-APC, ICAM1-FITC, Ly6G-FITC, Ly6G-PerCP/Cy5.5, LFA-1-PE, CD45-APC/Cy7, CD4-PE, CD8-PB, CXCR2-PerCP/Cy5.5, Gr1-PB, Ly6C-PB (BioLegend, San Diego, CA), CD11C-APC, CD11b-PECy7, CD3e PE/Cy7, CD3e-PerCP/Cy5.5, CD71-PE, F4/80-APC (eBioscience).

### Quantitative RT-PCR (qRT-PCR)

Total RNA from cells was isolated using RNeasy Mini kit (Qiagen, Valencia, CA). The quantity and quality of RNA were determined using a NanoDrop spectrophotometer (NanoDrop Inc., Wilmington DE). Primers and TaqMan probes (FAM dye-labeled) for *Il6, Tnfα, Arg1, Retnla, Nos2, Il12, Il10, and Hprt* were added to cDNA synthesized from total RNA with a High-Capacity cDNA Archive kit (Applied Biosystems, Carlsbad, CA). Product amplification was measured with an ABI HT7900 Fast real-time PCR system (Applied Biosystems). The threshold cycle (Ct) was obtained from duplicate samples and averaged. The ΔCt was the difference between the average Ct for the target gene and the housekeeping gene, *Hprt*. The ΔΔCt was the average ΔCt for a given sample point minus the average ΔCt of control (LFD) samples. The data are expressed as relative quantification calculated as 2^-ΔΔCt^.

### Cytokine measurements

Cytokines and chemokines concentrations for MCP-1/CCL2, KC/CXCL1, MIP-2/CXCL2, G-CSF, GM-CSF, and M-CSF were determined in plasma and/or broncho-alveolar lavage (BAL) samples using a magnetic bead luminex assay per manufactures instructions (R&D Systems, Minneapolis, MN) and analyzed on Bioplex 200 (Bio-Rad Laboratories, Redmond, WA).

### Statistics

Results are expressed as means ± SEM. Statistical significance was determined using Student’s t-test. Differences were considered significant if the *P*-value was <0.05.

## Results

### Diet-induced obesity is associated with an increase in monocytes and lymphocytes in the circulation at 3 months

C57Bl/6J male mice were maintained on a Western diet comprised of 42 % fat (HFD) for 3 months. At the time of harvest, standard chow control mice (LFD) weighed a mean of 31.2 ± 0.40 gm and the mice fed a Western diet weighed a mean of 46.3 ± 0.80 gm. We collected the blood from LFD and HFD groups at 3 months to quantify the circulating leukocyte subpopulations and their adhesion or chemokine receptors.

**Fig. 1:**
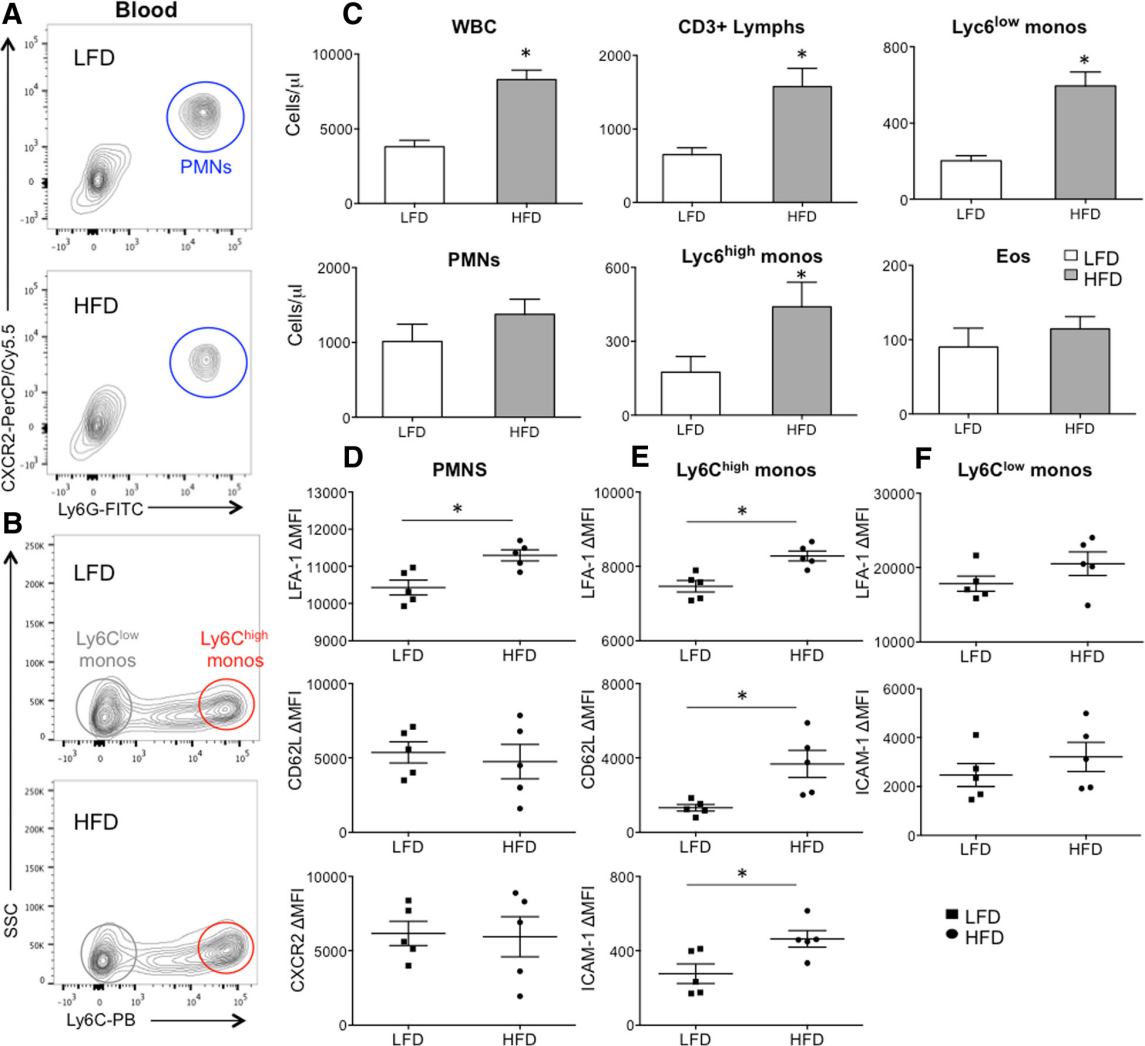
Circulating leukocytes after 3 months on a high fat (HFD) vs. low fat diet (LFD). Blood leukocytes were analyzed by FACS to quantify leukocytes subsets and their receptor expression. **a** Polymorphonuclear leukocytes (PMNs) were identified by Ly6G^pos^CXCR2^pos^ staining (*blue circle*). **b** After excluding PMNs, CD11b^+^SSC^low^ leukocytes were selected to identify Ly6C^low^ (*grey circle*) and Ly6C^high^ (*red circle*) monocytes (monos). **c** HFD mice had greater numbers of total white blood cells (WBCs) in the blood, comprised of lymphocytes (lymphs) and monocytes. **d** HFD PMNs expressed greater levels of LFA-1, but not CD62L or CXCR2. **e** HFD Ly6C^high^ monocytes expressed higher levels of LFA-1, CD62L and ICAM-1. **f** HFD Ly6C^low^ monocytes did not have a significant difference in LFA-1 or ICAM-1 expression. Mean fluorescent intensity (MFI). *p value < 0.05; mean ± SEM (*n* = 5 mice/group, experimental replicates 2)

In this compartment, HFD mice had an approximate 2 fold increase in total leukocytes (CD45^+^ cells), with significant increases in CD3^+^ lymphocytes and Ly6C^low^ and Ly6C^high^ monocytes (Ly6C^low^Ly6G^neg^CD11b^+^ cells and Ly6C^high^Ly6G^neg^CD11b^+^ cells) (Table 1; Fig. 1). Notably, at this time point, numbers of circulating neutrophils and eosinophils were similar between groups. Since leukocyte recruitment to tissue is dependent on expression of adhesion and chemokine receptor expression, we assessed expression of LFA-1, CD62L, ICAM-1, and CXCR2 on myeloid cells using FACS analysis. In the HFD group, circulating neutrophils (Ly6G^+^CD11b^+^CXCR2^+^SSC^high^) had greater expression of LFA-1 but no difference in CXCR2 or CD62L expression (Fig. 1a-c). ICAM-1 expression was not detected on a majority of blood PMNs (not shown). Ly6C^high^ monocytes (Ly6C^high^Ly6G^−^CD11b^+^ cells) from HFD mice expressed higher levels of CD62L, LFA-1, and ICAM-1 compared to those from LFD mice (Fig. 1d-f). Although the Ly6C^low^ monocyte population was significantly increased in the circulation of HFD mice, they had similar expression of LFA-1 and ICAM-1 between HFD and LFD groups (Fig. 1g, h) and did not express CD62L (not shown).

**Table 1:** Blood Cells at 3 months

Diet	WBC (cells/ul)a	PMNs	CD3+ Lymphocytes	Ly6c high Monocytes	Ly6c low Monocytes	Eosinophils
LFD	3.80 ± 0.39	1.01 ± 0.23	0.65 ± 0.08	0.17 ± 0.06	0.20 ± 0.06	0.09 ± 0.02
HFD	8.29 ± 0.57*	1.38 ± 0.20	1.57 ± 0.20***	0.44 ± 0.10***	0.59 ± 0.06**	0.11 ± 0.01

^a^ Data shown are number of cells (x10^3^) per microliter blood ± SEM

* *p* value <0.001, ** *p* value <0.005, *** *p* value <0.05

At this 3 month time point, we also harvested bone marrow cells from the femur to assess for changes in the myeloid cells in this compartment. Cellularity of the bone marrow was similar in both groups, with no difference in the myeloid populations (Table 2). We also assessed expression of adhesion receptors, LFA-1, CD62L, ICAM-1, and the neutrophil chemokine receptor, CXCR2. Within this compartment, the expression of these surface proteins on neutrophils and Ly6C^high^ monocytes was similar between the LFD and HFD groups (Additional file 1: Figure S1), indicating that changes in adhesion receptor expression occur outside the bone marrow. As was observed in the blood compartment, ICAM-1 was not expressed on a majority of the bone marrow PMNs (not shown).

**Table 2:** Bone Marrow Cells at 3 months

Diet	WBC (cells/ul)^a^	Neutrophils	Ly6c high Monocytes
LFD	23.6 ± 3.60	6.81 ± 0.50	3.68 ± 0.77
HFD	25.9 ± 3.20	6.96 ± 0.51	3.86 ± 0.73

^a^Data shown are number of cells (x10^6^) per milliliter ± SEM

### Diet-induced obesity is associated with evolving changes in leukocyte numbers and adhesion receptor expression at 3 and 6 months

**Fig. 2:**
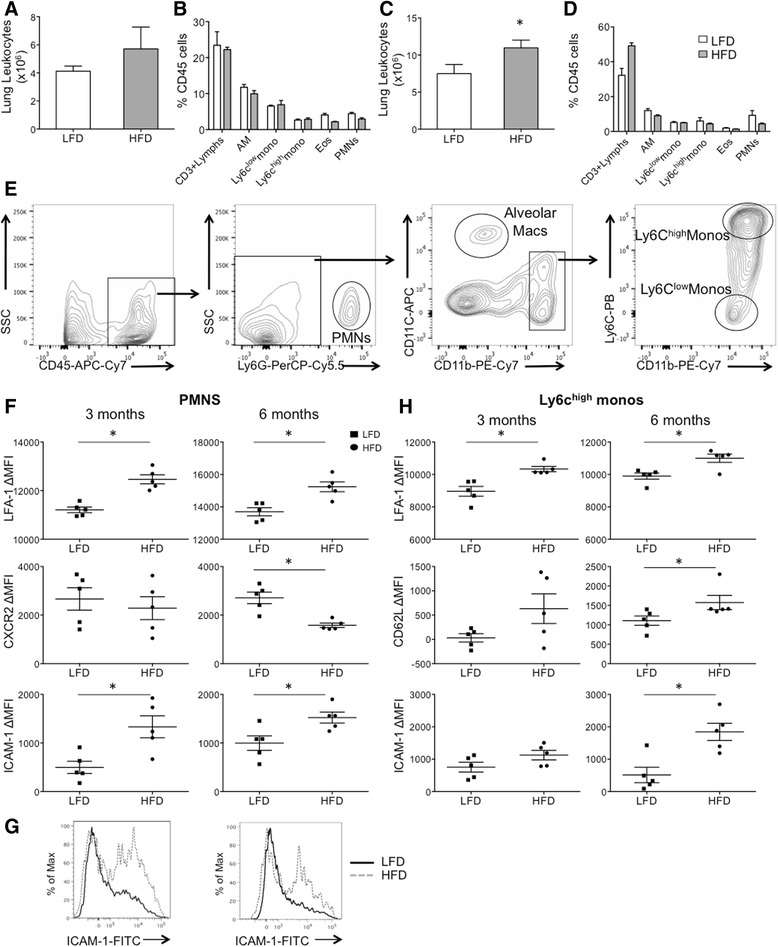
Adhesion Receptor Expression on Pulmonary Leukocytes at 3 Months. Leukocytes from perfused lungs were analyzed by FACS to quantify leukocyte subsets and their receptor expression. **a**, **b** At 3 months, there was a trend but no significant increase in total lung leukocytes in HFD mice. There was no significant change in leukocyte subsets at 3 months shown as % CD45^+^ cells. **c**, **d** By 6 months, there was a significant increase in total leukocytes isolated from the digested lung from HFD vs. LFD mice, and these changes were comprised of increases in CD3^+^ lymphocytes and Ly6C^low^ monocytes. **e** Gating strategy to identify lung PMNs, alveolar macrophages and monocyte populations. **f** PMN adhesion and chemokine receptor expression at 3 and 6 months demonstrate early and persistent increases in LFA-1 and ICAM-1 expression in HFD mice. CXCR2 expression was not significantly reduced until 6 months on a HFD. **g** Histograms for ICAM-1 expression demonstrate 2 PMN populations in the lung compartment, with an increase in the proportion of ICAM-1^+^ PMNs in HFD mice at 3 and 6 months. **h** Ly6C^high^ monocyte adhesion receptor expression at 3 and 6 months demonstrate early and sustained increase in LFA-1 expression and later increases in CD62L and ICAM-1 expression in HFD vs. LFD mice. Alveolar macrophage (AM), Eosinophils (Eos). *p value < 0.05; mean ± SEM (*n* = 5 mice/group, experimental replicates 3)

To assess the effects of HFD on the leukocyte populations in the naïve lung, we evaluated the leukocyte composition of vascular-perfused, unlavaged lungs at 3 and 6 months on the high and low fat diets. At 3 months, there was no difference in total leukocyte numbers or leukocyte subpopulations in the HFD group (Table 3, Fig. 2a, b). However, by 6 months, HFD mice had a significant increase in their pulmonary leukocytes (CD45^+^), comprised of increases in CD3^+^ lymphocytes and Ly6C^low^ monocytes (Table 4, Fig. 2c, d) indicating an evolving inflammatory phenotype with duration on a high-fat diet. Among the CD3^+^ lymphocytes, there was an increase in CD3^+^CD4^−^CD8^−^ lymphocytes and CD4^+^ but not CD8^+^ lymphocytes. In contrast, neutrophils (Ly6G^+^CD11b^+^F4/80^−^), eosinophils (CD11b^+^CD11c^−^SSC^high^), and alveolar macrophage (CD11c^+^CD11b^low^SSC^high^) numbers were unaltered by HFD.

**Table 3:** Lung Cells at 3 months

Diet	WBC (cells/ml)^a^	Neutrophils	CD3+ Lymphocytes	Ly6c high Monocytes	Ly6c low Monocytes	Eosinophils	Alveolar Macs
LFD	4.12 ± 0.36	0.18 ± 0.02	0.92 ± 0.16	0.17 ± 0.04	0.25 ± 0.13	0.16 ± 0.03	0.45 ± 0.03
HFD	5.71 ± 1.56	0.15 ± 0.03	1.21 ± 0.59	0.16 ± 0.05	0.37 ± 0.10	0.12 ± 0.04	0.50 ± 0.10

^a^Data shown are number of cells (x10^6^) per milliliter ± SEM

**Table 4:** Lung Cells at 6 months

Diet	WBC (cells/ml)*	Neutrophils	CD3+ Lymphocytes	Ly6c high Monocytes	Ly6c low Monocytes	Eosinophils	Alveolar Macs	CD3 + CD4-CD8-	CD3 + CD4+	CD3 + CD8+
LFD	7.51 ± 1.82	0.97 ± 0.33	2.46 ± 0.58	0.53 ± 0.15	0.45 ± 0.06	0.16 ± 0.04	0.82 ± 0.08	1.97 ± 0.49	0.49 ± 0.09	1.60 ± 0.21
HFD	11.0 ± 1.04	0.71 ± 0.09	5.46 ± 0.69+	0.54 ± 0.06	0.63 ± 0.06+	0.15 ± 0.03	0.98 ± 0.08	4.61 ± 0.65+	0.83 ± 0.05+	1.73 ± 0.11

* Data shown are number of cells (x10^6^) per milliliter ± SEM

+ *p* value <0.05

In addition to total leukocyte numbers and subpopulations, we also assessed the effect of the high fat diet on the expression of leukocyte adhesion and chemokine receptor expression on myeloid cells using gating strategies as shown to identify PMNs, alveolar macrophages and monocytes (Fig. 2e). At 3 months, HFD neutrophils isolated from the lung had greater LFA-1 and ICAM-1 expression but similar CXCR2 expression (Fig. 2f). CD62L, although expressed in the blood compartment PMNs, was not expressed by the pulmonary PMNs (not shown). Within the pulmonary compartment, 2 distinct neutrophil populations were present with a different distribution between HFD and LFD mice. These two neutrophil populations could be distinguished by absence or presence of ICAM-1 expression, and obese mice had a greater distribution of ICAM-1-expressing PMNs (56.4 ± 2.6 % vs. 68.7 ± 2.8 %, LFD vs. HFD, p value <0.01) (Fig. 2g). By 6 months, HFD neutrophils continued to express greater levels of LFA-1 and ICAM-1 and also expressed lower levels of CXCR2 (2710 ± 237 MFI vs. 1581 ± 90 MFI, LFD vs. HFD, p value <0.007) compared to those isolated from LFD mice (Fig. 2f). Hence, although PMN numbers were unchanged by HFD, there was an evolution of their surface markers with early upregulation of adhesion receptors and later downregulaton of their chemokine receptor, CXCR2.

At 3 months, HFD Ly6C^high^ monocytes isolated from the lung had greater LFA-1 expression and no difference in CD62L and ICAM-1 expression (Fig. 2h). However, by 6 months, LFA-1, CD62L, and ICAM-1 were all significantly increased in HFD Ly6C^high^ monocytes compared to their LFD controls (Fig. 2h). As in the circulation, we did not observe a change in adhesion receptor expression on Ly6C^low^ monocytes isolated from perfused lung at either time-point (not shown).

### Evolution of alveolar macrophage polarization markers in early and prolonged diet-induced obesity

**Fig. 3:**
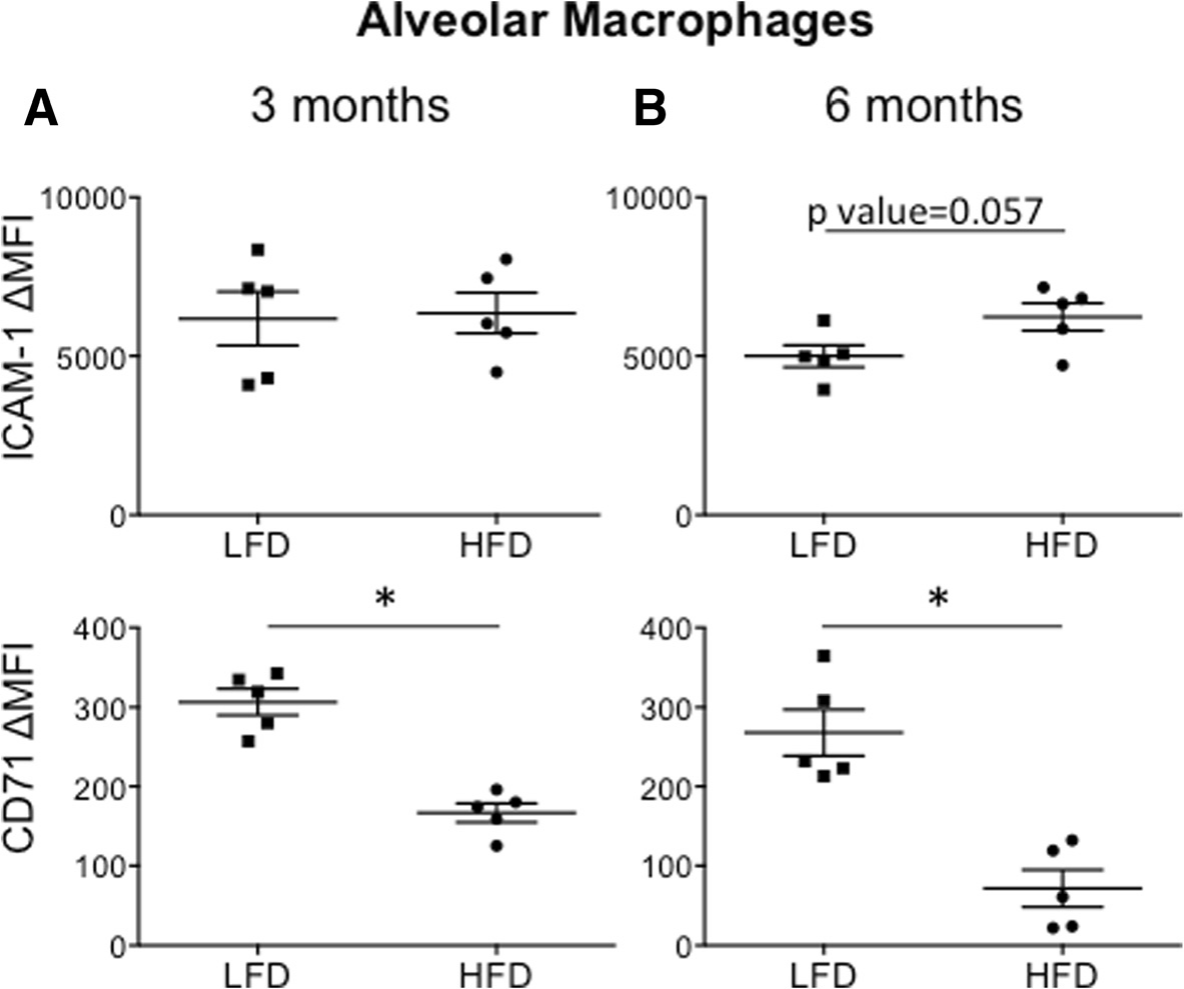
Alveolar Macrophage Polarization Markers at 3 and 6 Months. Alveolar macrophages were identified by FACS of whole lung digests. The surface expression for ICAM-1 and CD71 was assessed for changes in mean fluorescent intensity (ΔMFI) compared to isotype control. **a** At 3 months, there was no change in ICAM-1 expression but there was a significant reduction in CD71 expression in HFD mice. **b** By 6 months, there was an increase in ICAM-1 expression and a further reduction in CD71 expression in HFD mice. *p value < 0.05; mean ± SEM (*n* = 5 mice/group, experimental replicates 2–3)

Since obesity is associated with changes in macrophage numbers and polarization from M2 toward M1 cells in adipose tissue, we hypothesized that obesity would also alter the macrophage population in the lung. As reported above, alveolar macrophage numbers were similar in HFD and LFD groups. We assessed for differences in macrophage polarization markers in the resident alveolar macrophage. We previously showed that ICAM-1 is a marker of M1 activation, and CD71 is a marker of M2 activation on resident alveolar macrophages [14]. At 3 months, we observed reduced expression of the M2 marker, CD71, on the alveolar macrophage in the HFD group and no change in ICAM-1 expression (Fig. 3a). However, by 6 months, we saw both a reduction of CD71 and an increase in ICAM-1 expression in alveolar macrophages from the HFD group suggesting an evolution of macrophage polarization toward M1 or metabolically-activated cells associated with diet-induced obesity as has been reported in other tissues (Fig. 3b) [11, 12, 15].

### Altered LPS-induced myeloid recruitment in obese versus lean mice

**Fig. 4:**
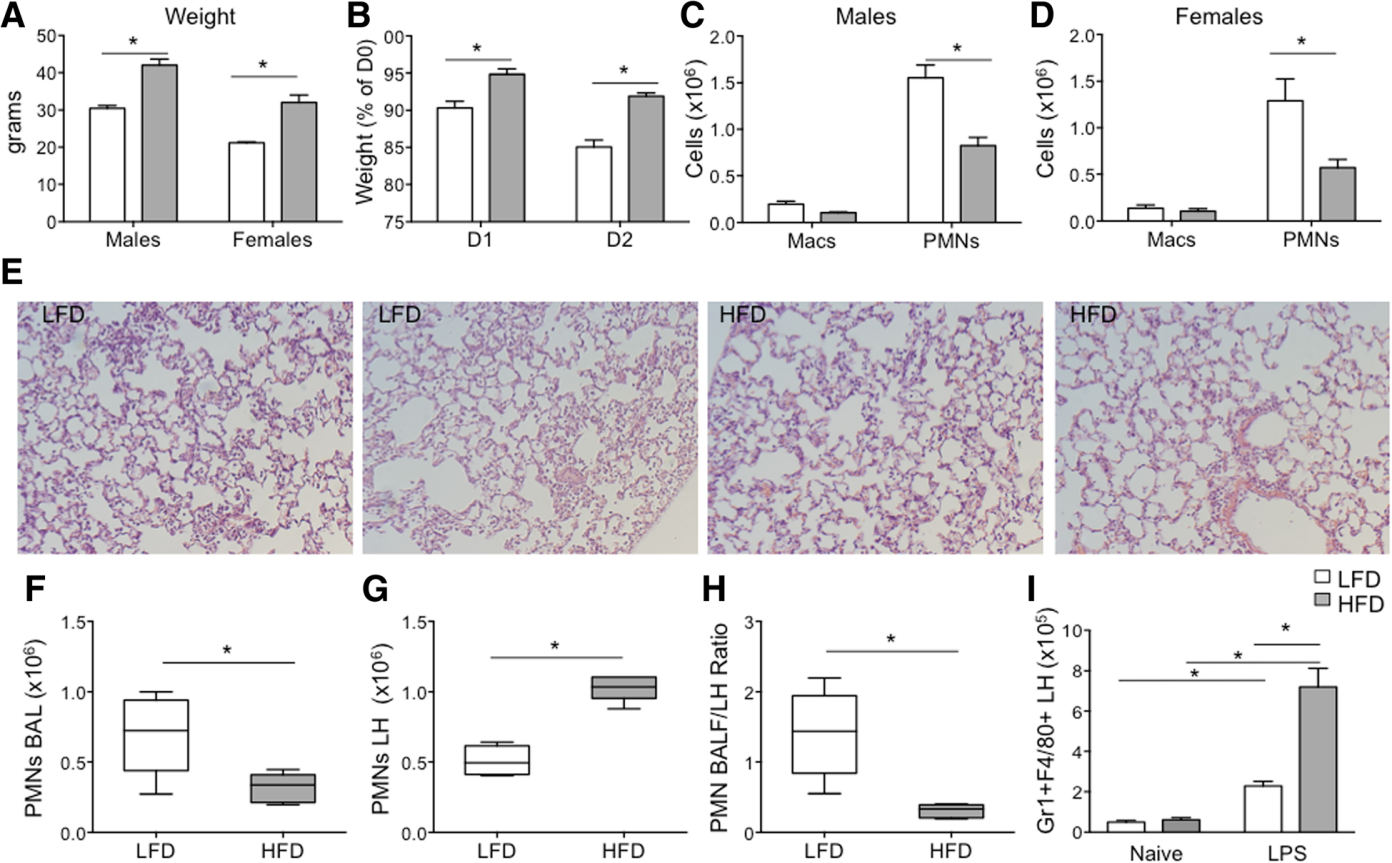
Inflammatory changes in the Lung and Alveolar Compartment Post-LPS. Male and female mice were maintained on a HFD or LFD for 3 months and then challenged with LPS intratracheally. Mice were evaluated at 24–48 h post-LPS. **a** Mean weight of male and female HFD and LFD mice at 3 months. **b** Mean weight (as a percentage of pre-LPS weight) at 24 h (D1) and 48 h (D2) post-LPS. The HFD group had reduced weight loss compared to LFD at both time-points. **c**, **d** Broncho-alveolar lavage macrophage (Macs) and neutrophil (PMNs) cell counts 24 h post-LPS instillation by gender. HFD male and female mice had reduced neutrophil recruitment into the alveolar space and similar numbers of macrophages compared to their respective LFD controls. **e** Representative histology of the lungs stained with hematoxylin and eosin from 2 LFD (left 2 panels) and 2 HFD (right 2 panels) mice 24 h post-LPS. Shown are the most severe areas of inflammation. **f**-**i** Broncho-alveolar lavage (BAL) and digested lung (LH) were analyzed by FACS for leukocyte distribution and counts at 24 h post-LPS. Boxplots show median with whiskers representing min-max values. **f**, **g** PMNs were reduced in the BAL compartment but significantly increased in the lung compartment from HFD mice. **h** The ratio of BAL/LH neutrophils was significantly reduced in LPS-treated HFD mice. **i** F4/80 + Gr1+ myeloid cells were significantly increased in the lung compartment of LPS-treated mice vs. naïve mice, with greater recruitment in LPS-treated HFD mice *p value < 0.05; mean ± SEM (*n* = 5 mice/group; experimental replicates 2–4)

To address if obesity alters the myeloid response to LPS-induced lung inflammation, we challenged obese and lean mice with LPS intratracheally to assess the acute inflammatory response in the alveolar and interstitial compartments in the lung. At 3 months, the average weight of males and females on the HFD were 37.7 % and 51.4 % greater than their respective LFD controls (Fig. 4a). After LPS exposure, HFD mice lost significantly less weight than similarly treated LFD mice (Fig. 4b). At 24 h, mice were euthanized and the inflammatory influx into the lung and alveolar compartment was assessed by broncho-alveolar lavage (BAL) and histopathology of lavaged lungs (Fig. 4c-e). BAL cell differential was determined using Diff-quik stained cytospins. Mice maintained on a HFD of either gender had reduced neutrophil influx in the alveolar compartment and similar numbers of alveolar macrophages (Fig. 4c-e). Total protein in the BALF, a marker of protein leak and lung injury, was not significantly reduced in the HFD groups (0.324 ± 0.04 vs. 0.265 ± 0.01 mg/mL, LFD vs. HFD; p value = 0.12), and inflammation by histology scoring demonstrated a modest decrease in LPS-treated HFD vs. LFD lungs (1.9 ± 0.2 vs. 2.4 ± 0.1; p value =0.05).

In a separate experiment, we assessed the inflammatory cell composition in the lung and alveolar compartment of LPS-treated mice using FACS with gating strategies outlined (Additional file 2: Figure S2). In these experiments, we both perfused the pulmonary vasculature and lavaged the alveolar compartment prior to lung digestion. Consistent with our previous experiment, we observed fewer neutrophils (Gr1^+^F4/80^−^CD11b^+^) in the BAL from obese versus lean mice as assessed by FACS (Fig. 4f). However, the neutrophil numbers were significantly increased in the lung compartment (LH) from LPS-challenged obese mice compared to lean mice (Fig. 4g). When expressed as a ratio of neutrophils in the BAL/LH compartments, there was nearly a 3 fold reduction in HFD neutrophil localization to the alveolar compartment, indicating a deficiency in neutrophil transmigration into the alveolar space (Fig. 4h). There were also increases in other myeloid cells (Gr1^+^F4/80^+^CD11b^+^) recruited to the lung compartment in HFD mice that were not present in significant numbers in naïve lungs, suggesting that diet-induced obesity also enhances the recruitment of subpopulations of macrophages to the injured lung (Fig. 4i).

**Fig. 5:**
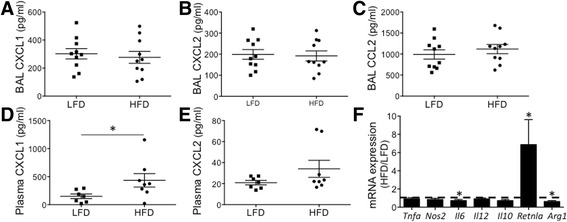
Chemokine gradients and inflammatory gene expression in LFD and HFD mice. The plasma and broncho-alveolar fluid from LPS-challenged LFD and HFD mice were analyzed for chemokine levels at 24 h. **a**-**c** CXCL1, CXCL2, and CCL2 levels in BAL from LPS-treated mice were similar in HFD and LFD mice. **d**, **e** CXCL1 but not CXCL2 was significantly increased in the plasma from LPS-treated HFD vs. LFD mice. **f** BAL cells were isolated at 24 h post-LPS, and mRNA assessed for gene expression using qRT-PCR. Data are expressed as a ratio of expression, HFD/LFD, and a ratio of 1 highlighted by the dashed line. *Il6* and *Arg1* expression was reduced in HFD mice. *Retnla* expression was increased in HFD mice, and *Tnfa, Nos2, Il12, Il10* were similarly expressed. *p value < 0.05; mean ± SEM (*n* = 7-10 mice/group)

Given differences related to macrophage and neutrophil recruitment, we assessed for differential expression of chemokines in the BAL and plasma of LPS-treated LFD and HFD mice. We did not observe differences in protein levels of neutrophil chemokines, CXCL1 and CXCL2 in the BAL compartment (Fig. 5a, b) or in the macrophage chemokine, CCL2 (Fig. 5c). However, there was increased CXCL1 in the plasma from LPS-treated HFD vs LFD mice indicating altered neutrophil chemokine gradients (Fig. 5d). We also assessed mRNA expression in inflammatory cells from the alveolar compartment of LPS-treated mice. Diet-induced obesity did not alter expression of *Tnfa, Nos2, Il12, Il10,* but was associated with a significant increase in *Retnla* expression and a small reduction in *Il6, and Arg1* expression (Fig. 5f).

## Discussion

There are pulmonary consequences to obesity, including increased prevalence of asthma, greater susceptibility to influenza, and possibility a reduced susceptibility to acute lung injury [5, 16–19]. Although it is well-established that obesity is associated with alterations of the immune system, little is known about how it alters the pulmonary immune cells to explain altered susceptibility or resistance to pulmonary injury. Recent studies implicate obesity-associated changes in innate lymphoid cells (ILCs) in asthma pathogenesis [20]. Lineage marker negative and Thy1.2, Sca-1, Rorγt and IL-7R positive ILC3 cells are increased in an IL-1β-dependent manner in obese mice, and more macrophages expressing IL-1β exist in these lungs. Our study did not examine these ILC populations, but we did find a progressive switch in macrophage polarization toward an M1 phenotype based on surface markers, along with an increase in monocyte numbers, which could represent important upstream changes of ILCs.

Our study also describes the changes associated with diet-induced obesity on other leukocyte subsets in the circulation and lung, including effects on adhesion receptor expression that may alter leukocyte recruitment or retention within the lung. We observed significant changes in adhesion receptor expression on blood and pulmonary neutrophils, Ly6c^high^ monocytes, and resident alveolar macrophages. With the exception of CD62L on alveolar macrophages, the HFD condition resulted in increases in ICAM-1, LFA-1, and CD62L in a cell-specific manner. LFA-1 is a ligand for ICAM-1, and leukocytes bind to endothelial cells via ICAM-1/LFA-1 [21]. In neutrophils deficient in LFA-1, neutrophil adhesion to unchallenged and LPS-challenged endothelium is impaired, highlighting its importance in neutrophil sequestration [22]. The neutrophil expression of LFA-1 was significantly increased in HFD neutrophils from the circulation and those localized to the lung. The mechanism of these increases is unknown, but interestingly, we did not observe differences in expression within the bone marrow compartment, suggesting that these changes occur within the circulation.

ICAM-1, can be up-regulated by IL-1β and other cytokines to promote leukocyte recruitment [23], and ICAM-1 up-regulation on leukocytes also serves as a marker of classically activated alveolar macrophages and more activated neutrophils [14, 24]. Based on ICAM-1 expression, we found two neutrophil populations sequestered in the naive lung. Similar to M1 and M2 nomenclature, neutrophil activation states have been classified as N1 and N2, with N1 cells having greater activation states [24]. ICAM-1 has been suggested as a marker of N1 cells, and the proportion of ICAM-1 positive neutrophils was increased in HFD lungs. Despite ICAM-1 detection on pulmonary PMNs, we did not detect ICAM-1 expression on BM or blood PMNs. Similarly, ICAM-1 expression has been reported to be low in circulating PMNs, and ICAM-1 up-regulation and CD62L down-regulation occur during endothelial cell adhesion and transendothelial cell migration (TEM) [24]. Hence, the observed increase in ICAM-1 and decrease in CD62L in the pulmonary vs. blood PMNs likely reflect changes associated with endothelial cell interaction/TEM. Furthermore, the augmented increase in ICAM-1^+^ PMNs in the naïve lungs from obese vs. lean mice may suggest differential PMN-endothelial cell interaction.

Post-LPS, we found that neutrophil recruitment into the alveolar space was impaired, similar to that reported by others [25]. However, our studies also found that neutrophil recruitment into the lung compartment was enhanced in LPS-treated obese mice. In addition to the differences in PMN adhesion receptor expression noted above, these changes were associated with more CXCL1 in the circulation but similar levels in the alveolar compartment levels, effectively reducing neutrophil chemokine gradient formation in HFD mice. Hence, the consequences of a HFD on the recruitment of PMNs in ALI may be multifactorial, and additional studies are necessary to examine the relative contribution of these factors.

Interestingly, we observed increased recruitment of other myeloid cells (Gr1^+^F4/80^+^CD11b^+^) in the lung associated with diet-induced obesity. These cells were not present in significant numbers in naïve mice, indicating that they are recruited to the lung during the inflammatory response to LPS. A majority of these cells localize to the lung and not the alveolar compartment. Although the mechanism for these obesity-associated differences is unknown, greater monocyte numbers in the circulation and altered adhesion receptors associated with obesity could contribute to these differences. Interestingly, these cells express surface markers of myeloid derived suppressor cells [26, 27] and obesity appears to be a key regulator of their presence in the inflamed lung. How these cells function in modulating lung injury or its resolution in obesity remain unexplored; however these cells have been shown to produce IL-10 and participate in resolution of lung injury [27].

In addition to the change in neutrophils and their markers of activation, we observed a similar change in alveolar macrophage polarization. A recent study describes a metabolically-activated macrophage phenotype in macrophages isolated from adipose tissue or bone marrow derived-macrophages stimulated with free fatty acids [15]. These cells down-regulate expression of some M2 markers, such as CD71, and up-regulate other M1 (IL-1β) and M2 genes. Similarly, we observed down-regulation of CD71 on alveolar macrophages, but up-regulation of the M2 gene, *Retnla* in the BAL cells recovered from LPS-treated obese mice. These findings suggest that obesity is capable of altering macrophage phenotypes beyond those within adipose tissue. Furthermore, alterations in macrophage programming in diet-induced obesity may reflect complex changes associated with metabolically-activated states, although our studies are limited by a narrow list of markers.

The drivers of the profound changes in lymphocytes and monocytes in the circulation and lung remain unknown, although leptin, which is increased in diet-induced obesity, is a known driver of lymphopoiesis and myelopoiesis [28, 29]. We examined the levels of other growth factors, GM-CSF and M-CSF in the serum and found no significant changes associated with obesity (not shown).

## Conclusions

In sum, we report on the profound inflammatory changes associated with diet-induced obesity in the unchallenged and LPS-challenged murine lung. Our results highlight the effects of diet-induced obesity on the murine blood and lung leukocyte populations, including increases in adhesion receptor expression that may contribute to altered recruitment or retention within the lung. Translation of these findings to people with obesity will be critical for determining the basic inflammatory underpinnings of pulmonary disease susceptibility. Our findings of an evolving inflammatory composition associated with the duration on a HFD suggest another important caveat and variable when using these models.

## Additional files

Additional file 1: Figure S1. Adhesion Receptor Expression on Bone Marrow Leukocytes at 3 Months on HFD vs. LFD. Bone marrow cells were isolated, counted, and immunostained with anti-murine antibodies to CD45, Ly6G, CD11b, Ly6C, CD62L, ICAM-1, CXCR2, and LFA-1 and analyzed by FACS. MFI for adhesion and chemokine receptors were measured in (A) PMNs and (B) Ly6C^high^ monocytes. (A) Among those receptors expressed by these cells, there was no difference in LFA-1, CD62L and CXCR2 levels in HFD vs. LFD PMNs. (B) Similarly, there was no difference in LFA-1 and CD62L expression in Ly6C^high^ monocytes. (TIF 1076 kb) Additional file 2: Figure S2. Gating strategy for BAL and LH leukocytes. Post-LPS, lungs were lavaged for BAL cells. The lungs were also perfused to remove vascular leukocytes and processed for flow cytometry. (A) BAL and (B) LH cells from LFD (top rows) and HFD (bottom rows) mice were gated based on size (FSC) and granularity (SSC). CD45^+^ leukocytes were selected, and alveolar macrophages identified as CD11c^+^GR1^−^ (black circle). Alveolar macrophages were also CD11b^low^SSC^high^. PMNs were identified as GR1^+^F4/80^−^CD11b^+^ cells (grey box). Another population of myeloid cells in the lung was identified as GR1^+^F4/80^+^CD11b^+^ (blue box). (TIF 4080 kb)
