# Supplementary figures and images for: Diet-induced obesity alters myeloid cell populations in naïve and injured lung
Source: Respir Res. 2016 Mar 8;17:24. doi: 10.1186/s12931-016-0341-8 (PMC4782295; doi:10.1186/s12931-016-0341-8)

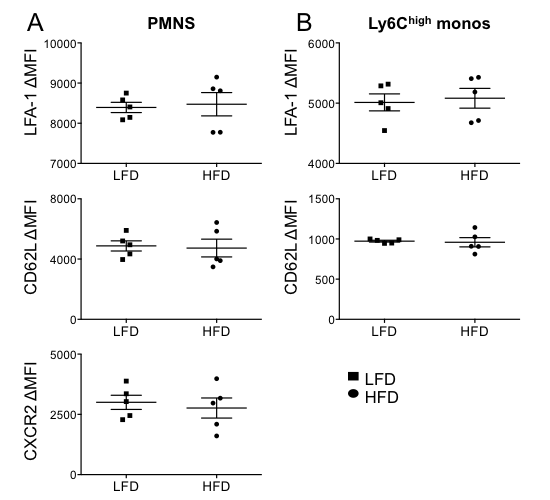

Supplement: Additional file 1: Figure S1. — Adhesion Receptor Expression on Bone Marrow Leukocytes at 3 Months on HFD vs. LFD. Bone marrow cells were isolated, counted, and immunostained with anti-murine antibodies to CD45, Ly6G, CD11b, Ly6C, CD62L, ICAM-1, CXCR2, and LFA-1 and analyzed by FACS. MFI for adhesion and chemokine receptors were measured in (A) PMNs and (B) Ly6Chigh monocytes. (A) Among those receptors expressed by these cells, there was no difference in LFA-1, CD62L and CXCR2 levels in HFD vs. LFD PMNs. (B) Similarly, there was no difference in LFA-1 and CD62L expression in Ly6Chigh monocytes. (TIF 1076 kb) [file 12931_2016_341_MOESM1_ESM.tif]

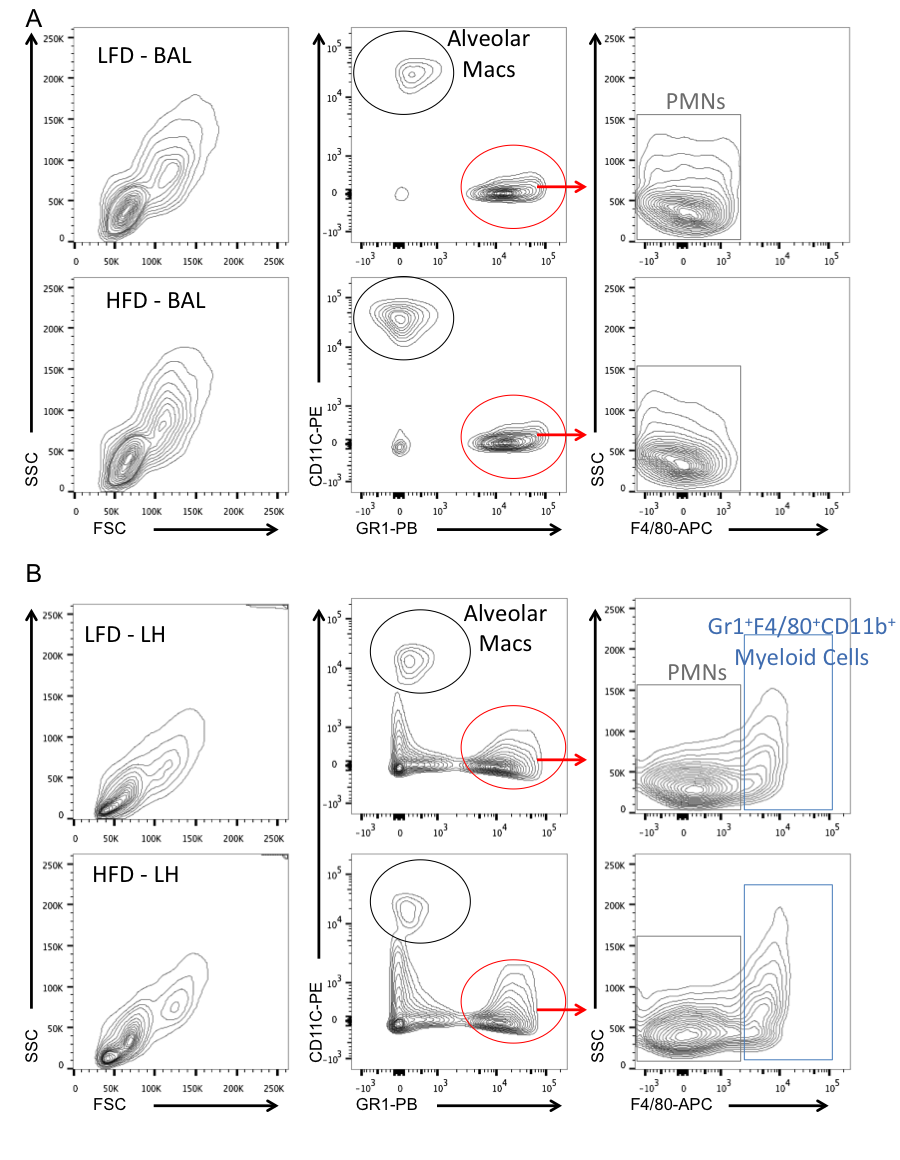

Supplement: Additional file 2: Figure S2. — Gating strategy for BAL and LH leukocytes. Post-LPS, lungs were lavaged for BAL cells. The lungs were also perfused to remove vascular leukocytes and processed for flow cytometry. (A) BAL and (B) LH cells from LFD (top rows) and HFD (bottom rows) mice were gated based on size (FSC) and granularity (SSC). CD45+ leukocytes were selected, and alveolar macrophages identified as CD11c+GR1− (black circle). Alveolar macrophages were also CD11blowSSChigh. PMNs were identified as GR1+F4/80−CD11b+ cells (grey box). Another population of myeloid cells in the lung was identified as GR1+F4/80+CD11b+ (blue box). (TIF 4080 kb) [file 12931_2016_341_MOESM2_ESM.tif]
